# RHDV 3C protein antagonizes type I interferon signaling by cleaving interferon promoter stimulated 1 protein

**DOI:** 10.1007/s11262-022-01958-w

**Published:** 2022-11-21

**Authors:** Yanjuan Men, Yonghui Wang, Hui Wang, Maoyin Zhang, Jing Liu, Yang Chen, Xufeng Han, Renjin Chen, Quangang Chen, Ankang Hu

**Affiliations:** 1 School of Life Science, Xuzhou Medical University, Xuzhou, Jiangsu China; 2 Kangda College of Nanjing Medical University, Lianyungang, Jiangsu China; 3 Cancer Institute, Xuzhou Medical University, Xuzhou, Jiangsu China; 4 Department of Anesthesiology, The Affiliated Hospital of Xuzhou Medical University, Xuzhou, Jiangsu China; 5 Department of Respiratory Medicine, Xuzhou Central Hospital, Xuzhou, Jiangsu China; 6 Laboratory Animal Center of Xuzhou Medical University, Xuzhou, Jiangsu China

**Keywords:** RHDV, Interferon, Innate immunity, Immune evasion

## Abstract

The host innate immune response to viral infection often involves the activation of type I interferons. Not surprisingly, many viruses have evolved various mechanisms to disable the interferon pathway and evade the antiviral response involving innate immunity. Rabbit hemorrhagic disease (RHD) is caused by RHD virus (RHDV), but whether it can antagonize the production of host interferon to establish infection has not been investigated. In this study, we found that during RHDV infection, the expressions of interferon and the interferon-stimulated gene were not activated. We constructed eukaryotic expression plasmids of all RHDV proteins, and found that RHDV 3C protein inhibited poly(I:C)-induced interferon expressions. Using siRNA to interfere with the expressions of TLR3 and MDA5, we found that the MDA5 signal pathway was used by the 3C protein to inhibit poly(I:C)-induced interferon expression. This effect was mediated by cleaving the interferon promoter stimulated 1 (IPS-1) protein. Finally, our study showed that interferon was effective against RHDV infection. In summary, our findings showed that the RHDV 3C protein was a new interferon antagonist. These results increase our understanding of the escape mechanism from innate immunity mediated by the RHDV 3C protein.

## Introduction

Rabbit hemorrhagic disease virus (RHDV) was first discovered in China in 1984 [1]. It has subsequently spread worldwide within a few years, resulting in great economic losses in the rabbit industry. RHDV belongs to the genus *Lagovirus europaeus*, family Caliciviridae [2], and the virions are non-enveloped and icosahedral caliciviruses [3]. RHDV contains a 7,437-nucleotide positive sense single-stranded genomic RNA, which is composed of two slightly overlapping open reading frames (ORFs) and a 2.2-kb designated subgenomic RNA [4]. ORF1 encodes a large protein cleaved by a virus-encoded protease into the main capsid protein of RHDV VP60 and seven mature non-structural proteins (p16, p23, helicase, p29, Vpg, protease, and RdRp) [5, 6], with ORF2 encoding another structural protein (VP10) [7, 8].

RHDV infection could be controlled by biosecurity and vaccination. The main vaccine used to prevent and control RHDV is inactivated virus. However, no suitable immortalized cell line has been able to culture RHDV in vitro. RHDVs are all purified from artificially infected rabbit liver tissue, so the antigen purification is difficult and costly, and the current vaccines are unacceptable from both safety and ethical perspectives [6].

Type I interferons are known as a type of primary cytokine, which protect against viral infection [9]. During virus infection, pattern recognition receptors recognize viral nucleic acids, sugars, lipoproteins, or other small molecules, and induce innate immune responses [10]. Toll-like receptor 3 (TLR-3) can recognize dsRNA and recruit TIR domain-containing adaptor-inducing interferon-β (TRIF), which then activates IκB kinase and TANK binding kinase-1 (TBK-1), then IRF-3 and NF-κB are activated, finally leading to type I interferon expression [11]. RIG-I/MDA5 can also recognize dsRNA, and its caspase-recruiting domains interact with interferon promoter stimulated 1 (IPS-1). Similar to TLR-3, IPS-1 mediates activation of TBK-1 and IKKε, which stimulate the expression of type I interferons [12]. Not surprisingly, many viruses have evolved various strategies to evade immune surveillance using targeted molecules. Cleavage of the crucial signal molecules is an effective escape method, because it can effectively disrupt the signal transduction to inhibit interferon production. For example, Coxsackievirus B, enterovirus 71 virus, and hepatitis A virus can inhibit interferon by cleavage of IPS-I and TRIF to inhibit antiviral responses [13].

This study therefore identified the molecular mechanism of RHDV, which regulated the production of type I interferon, and showed that RHDV could not induce the production of interferon-β in rabbits after infection, even during the later stages of infection; but RHDV could inhibit the expression of interferon-β. Furthermore, we found that the RHDV-encoded 3C protein participated in interferon-β repression, by cleaving IPS-1 and inhibiting the RIG-I/MDA5 signal pathway. Finally, in vivo experiments in rabbits were used to verify that RHDV evasion of host innate immunity depended on its inhibition of interferon. We found that poly(I:C)-induced interferon production protected rabbits against RHDV infection.

## Materials and methods

### Cell culture and virus infection

RK-13 cells were cultured in DMEM (Gibco, Waltham, MA, USA) supplemented with 10% heated-inactivated fetal bovine serum, 100 U/mL penicillin, and 100 μg/mL streptomycin sulfate at 37 °C in a 5% CO_2_ incubator. RHDV was isolated from livers of rabbits infected with RHDV. Briefly, Liver tissues were collected, and homogenized in PBS. The suspensions were centrifugated at 5000 rpm for 10 min, after that the clear supernatants were collected and kept at -80℃ till used. New Zealand white rabbits were used in this study. The experiment was approved by the Laboratory Animal Ethics Committee of Xuzhou Medical University (No. 201911A089). Rabbits were infected with RHDV by subcutaneous injection.

### ELISA

Six rabbits were infected with RHDV, and venous blood was collected after 12 h, 24 h, 36 h, and 48 h, and stored at 4 °C. After blood clotting, rabbits were euthanized, and blood samples were centrifuged at 800 rpm for 5 min. The upper serum was collected and the interferon-β concentration was evaluated by an ELISA according to the manufacturer’s instructions (LifeSpan Biosciences, Seattle, WA, USA).

### Western blotting

RK-13 cells were seeded in 6 well plates. When cells grown to 60% confluence, cells were transfected with plasmids (2 μg). Before protein collection, the cells were washed with cold phosphate-buffered saline (PBS), and then lysed with NP40 lysis buffer. After incubation for 10 min on ice, the lysates were harvested using 5 × sodium dodecyl sulfate–polyacrylamide gel electrophoresis (SDS-PAGE) sample buffer. Equal amounts of proteins from cell lysates were then resolved using 12% SDS-PAGE and transferred to polyvinylidene difluoride membranes (Millipore, Bedford, MA, USA). Densitometry analysis was performed using ImageJ software (National Institutes of Health, Bethesda, MD, USA) and band intensities were normalized to those of β-actin. Antibodies specific for HA and Flag were purchased from ProteinTech (Wuhan, China).

### Construction of recombinant plasmids

According to the cleavage site of the ORF1-encoded protein of RHDV published by Uniport and the ORF2 sequence in the Genebank (Accession number HM623309), the primers were designed to amplify all encoded proteins of RHDV (pCAGGS-p16, pCAGGS-p23, pCAGGS-NTPase, pCAGGS-p41, pCAGGS-p23/2, pCAGGS-p29, pCAGGS-p18, pCAGGS-VPg, pCAGGS-3C, pCAGGS-RdRp, pCAGGS-VP60, and pCAGGS-VP10), then cloned into the pCAGGS vector with an N-terminal HA tag. Rabbit IPS-1 was subcloned into the pCAGGS vector and with an N-terminal FLAG tag. Primers are listed in Table 1.

**Table 1:** Primers and siRNAs sequence

Primer	Sequence 5′ → 3′
P16-F	TTTGAATTCATGGCGGCTATGTCGCGCCTTACT
P16-R	GCGGTCGACTTATTCAAAAATAGGGGTGGGAAA
P23-F	ATAGAATTCATGGGGGAGGTTGACGACTTATTT
P23-R	GCGGTCGACTTATTCAATGATCAAGTTGACAGT
NTPase-F	ATAGAATTCATGGGTGTGAAGAGTTTCTGGGAC
NTPase-R	ATAGTCGACTTACTCGAATGAGGCCACGTCAGG
P41-F	ATAGTCGACATGCCTGACGTGGCCTCATTCGAG
P41-R	GCGGGATCCTTATCATAGTCATTGTCATAAAAG
P23/2-F	GAGGTCGACTTAGTCAACGTTGTCTGATGCCAC
P23/2-R	GAGGTCGACTTAGTCAACGTTGTCTGATGCCAC
P29-F	GCGGAATTCATGGGTGCCAACAAATTTAACTTT
P29-R	GAGGTCGACTTACTGAAAAGCCTTCGTTGCAAC
P18-F	ATAGTCGACATGCGCGGTGACCAAGGCGTTGAC
P18-R	GCGGGATCCTTACTCATAGTCATTGTCATAAA
VPg-F	GAGGTCGACATGGGCGTGAAAGGCAAGACAAAA
VPg-R	GCGGGATCCTTACTCATAGTCATTGTCATAAAAG
3C-F	GAGGAATTCATGGGTTTACCTGGGTTCATGAGA
3C-R	GCGGTCGACTTATTCATAAACTCCCTTTGTAAT
RDRP-F	GCGGAATTCATGACGTCAAACTTCTTCTGTGGT
RDRP-R	GCGGTCGACTTACTCCATAACATTCACAAATTC
VP60-F	TATGAATTCATGGGCAAAGCCCGTGCAGCGC
VP60-R	TATGTCGACTTAGACATAAGAAAAGCCATTGGC
ORF2-F	GCGGTCGACATGGCTTTTCTTATGTCTGAATTC
ORF2-R	TATGGATCCTTAAACACTGGACTCGCCAGTGGT
TLR3-F	ATGCACGGTGAGACAGGAAG
TLR3-R	GGCTCCAGCTTTGAGATGGT
MDA5-F	AAGCCAGTGATTGCCCTACC
MDA5-R	TGGATCCTCTCGGGTGTCAT
IFN-β-F	TGGAACGACTGAGGACTGCC
IFN-β-R	AGGGTCTCATTCCAGCCAGTG
GAPDH-F	AGACACGATGGTGAAGGTCG
GAPDH-R	TGCCGTGGGTGGAATCATAC
siTLR3-F	ACUCAUUCCCAUUGUUUUCUG
siTLR3-R	GAAAACAAUGGGAAUGAGUCA
siMDA5-F	AGGUAUUUGAGUCAACUUCAG
siMDA5-R	GAAGUUGACUCAAAUACCUGA

### RNA extraction and qRT-PCR

The rabbits were injected subcutaneously at the neck with 10 times LD_50_ RHDV or PBS. Total RNAs were extracted from liver tissue or RK-13 cells using TRIzol reagent (Invitrogen, Carlsbad, CA, USA) and reverse-transcribed (1 μg) into cDNA using avian myeloblastoma virus reverse transcriptase (Takara, Shiga, Japan). The qRT-PCR was conducted using a Light Cycler 480 (Roche, Basel, Switzerland) using SYBR Green I Master (Roche).

### Statistical analysis

All experiments were conducted in triplicate, and data are presented as the mean ± standard deviation (SD). A *P* value of < 0.05 was considered significant, and *P* < 0.01 was considered highly significant.

## Results

### RHDV infection could not induce the interferon-stimulated gene (ISG) and type I interferon expression

**Fig. 1:**
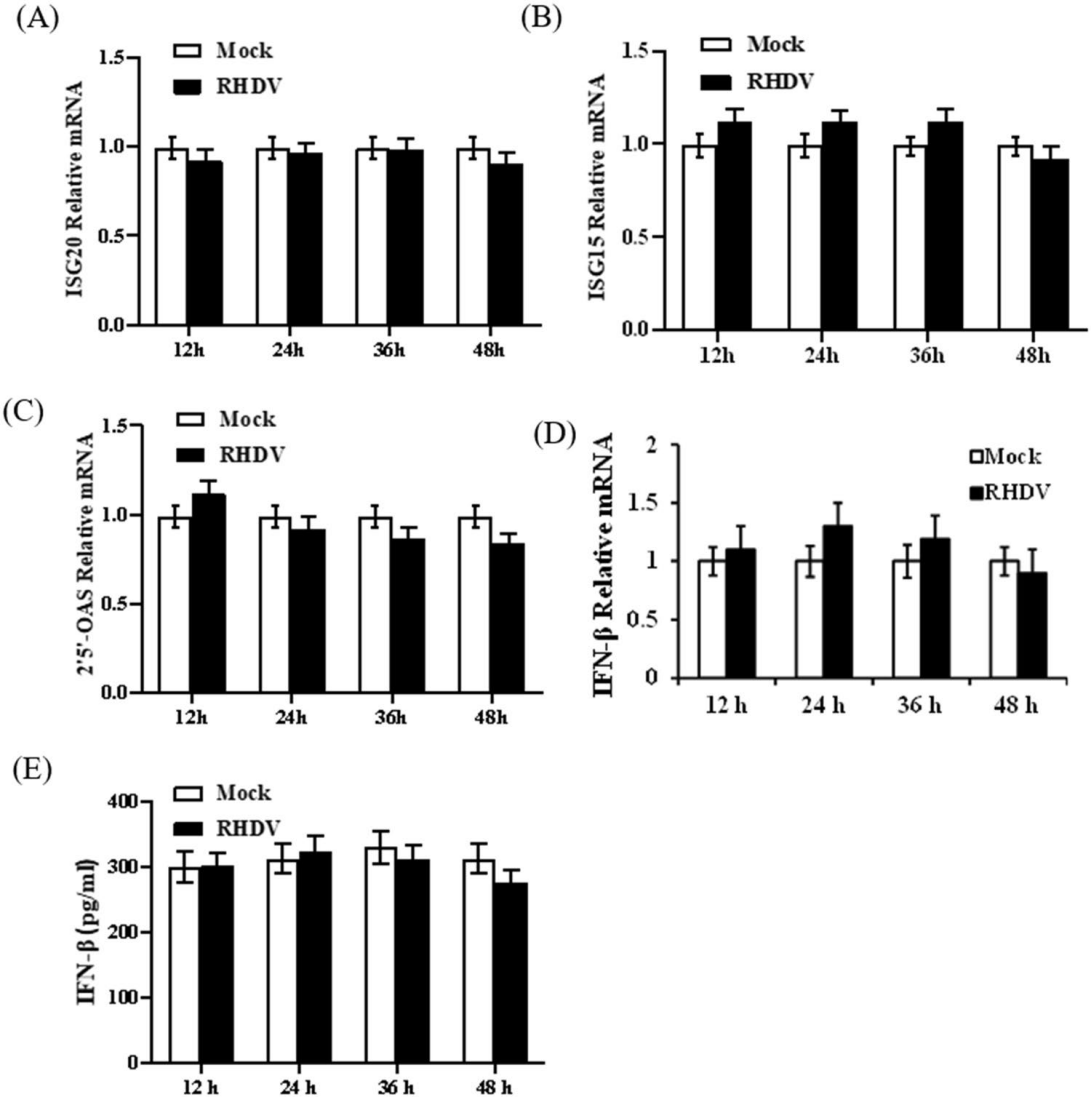
Interferon-β expression was not activated during RHDV infection. Rabbits were infected with RHDV for 24 h, then liver tissue and venous blood were collected. **A**–**D** The qRT-PCR was used to detect the mRNA expression levels of ISG15, ISG20, 2′5′-OAS, and interferon-β in liver tissue. **E** ELISA was used to detect the expression of interferon-β in venous blood of rabbits infected with RHDV. The results are presented as the mean ± SD. *P < 0.05, **P < 0.01

ISGs can directly inhibit virus infection. Hence, we first detected ISG expression during RHDV infection. The rabbits were euthanized, and liver tissues were extracted at 12 h, 24 h, 36 h and 48 h post-infection. Total RNA was extracted, and the expressions of ISGs (ISG15, ISG20, and 2′5'-OAS) were detected by qRT-PCR. We found that ISG mRNA levels remained unchanged at all time points (Fig. 1A, 1B, 1C). The ISG expressions were generally activated by interferon. To determine the reason why ISG expressions were not activated after RHDV infection, we determined the expressions of interferon-β. Changes in the expression of interferon-β were detected by qRT-PCR and ELISA, which showed that RHDV infection failed to induce the production of interferon-β throughout the infection time course (Fig. 1D, E).

### RHDV 3C protein antagonized type I interferon expression during RHDV infection

**Fig. 2:**
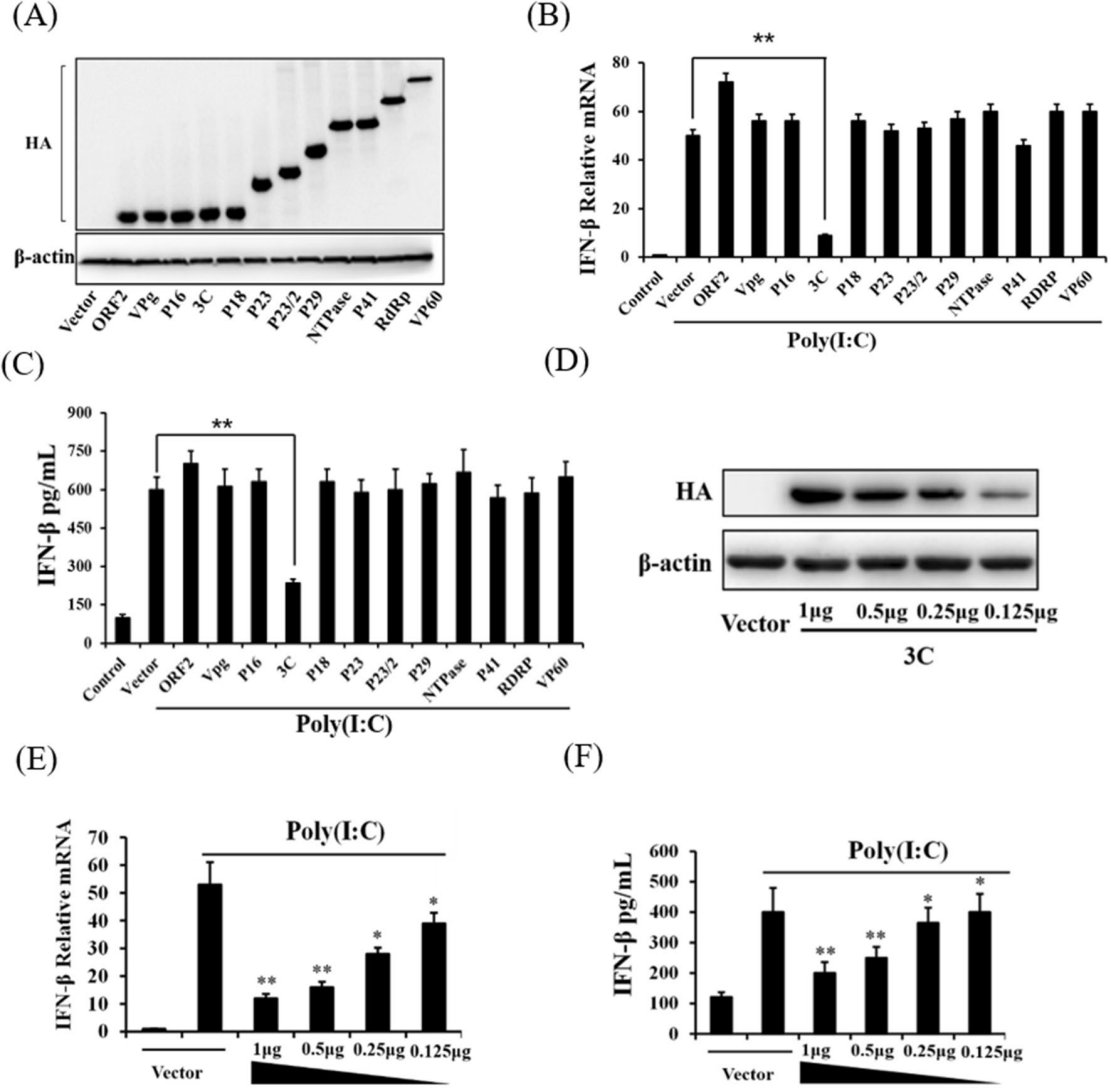
RHDV 3C protein inhibits poly(I:C) induced interferon-β expression. The eukaryotic expression plasmid of RHDV proteins were transfected into RK-13 cells 24 h later. **A** The expressions of RHDV proteins were determined by western blotting. **B**, **C** The cells were transfected with poly(I:C) for 12 h, qRT-PCR was used to determine the levels of mRNA expression, and ELISA was used to detect the protein expression levels of interferon-β in the supernatant. **D**–**F** RK-13 cells were transfected with various amounts of expression vectors encoding RHDV 3C (1 μg, 0.5 μg, 0.25 μg, and 0.125 μg), and 24 h later, the cells were transfected with poly(I:C) for 12 h. Western blotting verified the overexpression of 3C protein at different doses. The qRT-PCR was used to determine the levels of mRNA expression. ELISA was used to detect the expression levels of interferon-β protein. The results are presented as the mean ± SD. *P < 0.05, **P < 0.01

A previous study reported that rabbits inoculated with RHDV vaccine induced interferon activation [14]. In the present study, we found that RHDV infection did not have this effect, so we speculated that the RHDV-encoded protein might play a role in antagonizing interferon expression. To determine how RHDV infection influenced type I interferon expression, we constructed eukaryotic expression plasmids of all RHDV proteins, and transfected them into RK-13 cells. We found that all the RHDV proteins could be successfully expressed (Fig. 2A). Poly(I:C) was used to activate interferon production. The expression of interferon-β in the supernatant was evaluated using an ELISA, and mRNA expression of interferon-β was detected using qRT-PCR. The results showed that poly(I:C) significantly induced the expression of interferon-β, but this induction was significantly inhibited in cells overexpressing RHDV 3C protein (Fig. 2B, C). Finally, we overexpressed 3C protein at different doses, and found that this protein inhibited poly(I:C)-induced interferon-β expression in a dose-dependent manner (Fig. 2D–F).

### The 3C protein inhibited the expression of interferon-β through the MDA5 pathway

**Fig. 3:**
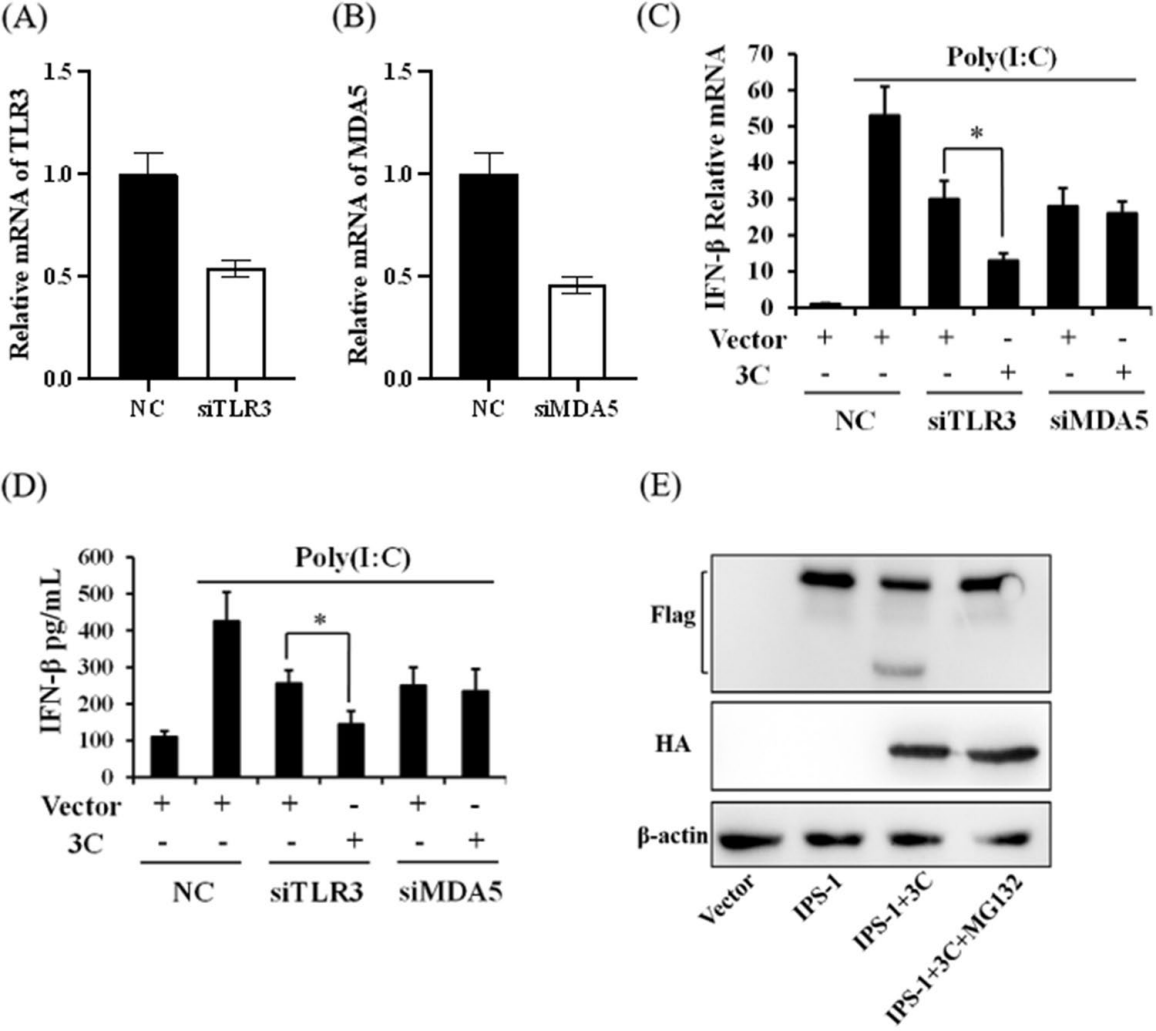
The 3C-like protein inhibits the expression of interferon-β by cleaving IPS-1. **A** and **B** RK-13 cells were transfected with siRNA targeted to TLR3 or MDA5, and 24 h later, qPCR was performed to detect the expressions of TLR3 and MDA5. **C** and **D** RK-13 cells were transfected with siRNA-TLR3, siRNA-MDA5, or negative control (NC), and 24 h later, the cells were transfected with poly(I:C) for another 24 h. The cells and supernatant were collected, and mRNA and protein expression levels were determined using qRT-PCR and an ELISA. **E** RK-13 cells were co-transfected with rabbit IPS-1(Flag tag) and a RHDV 3C protein (HA tag) expression plasmid, and then the cells were treated with MG132 or dimethyl sulfoxide for 24 h. The cleavage effect of 3C protein on IPS-1 was then detected by western blotting. All results are presented as the mean ± SD, *P < 0.05

Virus RNA is mainly recognized by TLR3 and MDA5, and induces the production of interferon by MyD88/TRIF or IPS-1. To determine which pathway was used by RHDV to suppress type I interferon expression, siRNA of TLR3 or MDA5 was transfected into RK-13 cells to disrupt these two signal pathways. We found that siRNA significantly and specifically inhibited mRNA expression (Fig. 3A, B). In parallel experiments, we found that either knockdown of TLR3 or MDA5 alleviated poly(I:C)-induced interferon-β expression. However, 3C protein further inhibited interferon-β expression only in TLR3 knockdown cells (Fig. 3C, D). Together, the results showed that the 3C protein might disrupt the MDA5 signal pathway to inhibit activation of interferon.

Viruses can inhibit interferon expression through various mechanisms. To determine how RHDV influenced the MAD5 signal pathway, we characterized the expressions of downstream proteins, and found that the IPS-1 protein in the control group had only one band; however, during overexpression of 3C protein, another band with a molecular weight smaller than normal IPS-1 was detected, so we proposed that the 3C protein cleaved IPS-1 (Fig. 3E). To further confirm whether this cleavage depended on proteasome activity, MG132 was used to inhibit the proteasome, which showed that MG132 inhibited cleavage activity of the RHDV 3C protein (Fig. 3E).

### RHDV evasion of host innate immunity depended on its inhibition of interferon induction

**Fig. 4:**
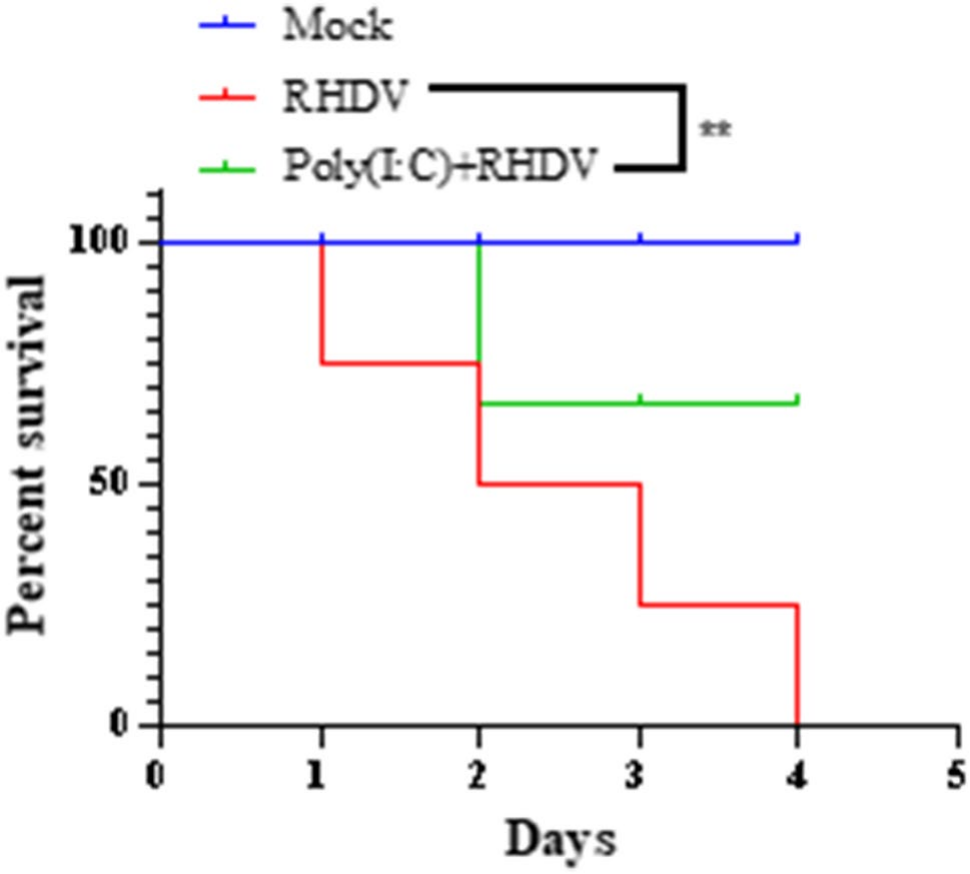
RHDV evades host innate immunity by inhibiting interferon expression. Rabbits were injected with ploy(I:C) for 24 h, and then inoculated with RHDV, followed by determinations of survival. **P < 0.01

To verify whether the immune evasion of RHDV depended on its inhibition of interferon, 24 rabbits were randomly divided into three groups: rabbits infected with RHDV, rabbits mock-infected with RHDV, and rabbits pretreated with poly(I:C) (500 μg/kg) before infection with RHDV. After inoculation with RHDV, the overall survival was recorded. Figure 4 shows that induction of interferon expression increased protection of rabbits against RHDV infection.

## Discussion

During viral infection, the innate immune system is activated, leading to the induction of pro-inflammatory factors, interferon, and other cytokines to protect against virus infection. Among them, interferon and interferon-induced cellular antiviral response are primary defense mechanisms [9, 10]. Type I interferon binds to cell surface receptors and triggers the induction of hundreds of ISGs, which resist viral replication [15, 16]. However, in the present study, during RHDV infection, there was no induction of ISG expression, so we speculated that interferon activation might have been inhibited during RHDV infection. Indeed, neither mRNA nor interferon-β was activated in RHDV-infected rabbits.

Many viruses have developed different strategies to escape innate immunity and replicate [16–18]. For example, influenza A virus encodes nonstructural protein 1, which prevents induction of the interferon-β promoter by inhibiting the activation of transcription factors [19]. In addition, poliovirus-induced cleavage of MDA-5 may be a mechanism to antagonize production of type I interferon [20], and porcine reproductive and respiratory syndrome virus infection inhibits interferon-β expression by negative regulation of IRF-3 phosphorylation [21]. To address the immunosuppressive mechanism of RHDV, we constructed eukaryotic expression plasmids of each RHDV protein and transfected them into RK-13 cells, while poly(I:C) was transfected into cells to active interferon expression. We found that poly(I:C) significantly induced the expression of interferon-β, but this induction effect was significantly inhibited in cells overexpressing RHDV 3C protein.

In the process of RNA virus infection, the activation of interferon mainly involves two pathways: TLR3 and RIG-I/MDA5. Poly(I:C) is mainly recognized by TLR3 and MDA5, and induces the production of interferon by MyD88/TRIF or IPS-1, respectively. To determine which pathway the 3C protein used to inhibit the production of interferon, we used TLR3 and MDA5 interference molecules to block the two pathways, and further tested the effect of the 3C protein on interferon expression induced by poly(I:C). We found that 3C protein impaired poly(I:C)-induced interferon-β expression, and knockdown of MDA5 did not further suppress interferon-β expression; however, the inhibition effect was further blocked by siTLR3, so the 3C protein might influence the MDA5 signal pathway.

Virus replication and assembly require encoded proteases to cleave the translated polyprotein into mature structural and non-structural proteins. At the same time, these virus-encoded proteases often target cellular proteins to evade the host innate immune surveillance. For example, during foot-and-mouth disease and encephalomyocarditis virus infections, the immune response is blocked by 3C-like proteases encoded by the viruses by cleaving the NEMO, RJG-I, and MDA5 host proteins [22, 23]. In addition, these proteases can inhibit host cell cap-dependent translation by cleaving translation initiation factors. Based on these considerations, we determined the effect of 3C protein cleavage on proteins of the MDA5 pathway. When detecting the expression of IPS-1 by western blotting, there was only one band in the control cells; however, another faster-migrating protein band was observed in overexpression of 3C protein cells, confirming the cleavage effect of 3C protein on IPS-1. This cleavage effect was blocked by MG132, confirming that 3C protein cleavage depended on its proteinase activity. Finally, we found that poly(I:C) injection to induce interferon expression significantly protected against RHDV infection.

In summary, we demonstrated that during infection, RHDV-encoded 3C protein antagonized interferon production by cleaving IPS-1 and altering the innate immunity. Our study clearly revealed the immunosuppressive mechanism, so a better understanding of the interactions between RHDV and the interferon signaling pathway will help in the development of novel therapeutic targets and more effective vaccines.

## References

[1] Abrantes J, van der Loo W, Le Pendu J, Esteves PJ (2012). Rabbit haemorrhagic disease (RHD) and rabbit haemorrhagic disease virus (RHDV): a review. Vet Res.

[2] Xu ZJ, Chen WX (1989). Viral haemorrhagic disease in rabbits: a review. Vet Res Commun.

[3] Parra F, Boga JA, Marin MS, Casais R (1993). The amino terminal sequence of VP60 from rabbit hemorrhagic disease virus supports its putative subgenomic origin. Virus Res.

[4] Meyers G, Wirblich C, Thiel HJ (1991). Rabbit hemorrhagic disease virus–molecular cloning and nucleotide sequencing of a calicivirus genome. Virology.

[5] Meyers G, Wirblich C, Thiel HJ, Thumfart JO (2000). Rabbit hemorrhagic disease virus: genome organization and polyprotein processing of a calicivirus studied after transient expression of cDNA constructs. Virology.

[6] Zhu J, Miao Q, Tan Y, Guo H, Liu T, Wang B, Chen Z, Li C, Liu G (2017). Inclusion of an Arg-Gly-Asp receptor-recognition motif into the capsid protein of rabbit hemorrhagic disease virus enables culture of the virus in vitro. J Biol Chem.

[7] Chen L, Liu G, Ni Z, Yu B, Yun T, Song Y, Hua J, Li S, Chen J (2009). Minor structural protein VP2 in rabbit hemorrhagic disease virus downregulates the expression of the viral capsid protein VP60. J Gen Virol.

[8] Wirblich C, Thiel HJ, Meyers G (1996). Genetic map of the calicivirus rabbit hemorrhagic disease virus as deduced from in vitro translation studies. J Virol.

[9] Randall RE, Goodbourn S (2008). Interferons and viruses: an interplay between induction, signalling, antiviral responses and virus countermeasures. J Gen Virol.

[10] Kawai T, Akira S (2011). Toll-like receptors and their crosstalk with other innate receptors in infection and immunity. Immunity.

[11] Park GB, Hur DY, Kim YS, Lee HK, Yang JW, Kim D (2015). TLR3/TRIF signalling pathway regulates IL-32 and IFN-beta secretion through activation of RIP-1 and TRAF in the human cornea. J Cell Mol Med.

[12] Nasirudeen AM, Wong HH, Thien P, Xu S, Lam KP, Liu DX (2011). RIG-I, MDA5 and TLR3 synergistically play an important role in restriction of dengue virus infection. PLoS Negl Trop Dis.

[13] Mukherjee A, Morosky SA, Delorme-Axford E, Dybdahl-Sissoko N, Oberste MS, Wang T, Coyne CB (2011). The coxsackievirus B 3C protease cleaves MAVS and TRIF to attenuate host type I interferon and apoptotic signaling. PLoS Pathog.

[14] Yanli Zheng KW, Ma H, Gao Y (2013). Research progress of rabbit viral hemorrhagic disease vaccine. Progress in Veterinary Medicine.

[15] Teijaro JR (2016). Type I interferons in viral control and immune regulation. Curr Opin Virol.

[16] Alcami A, Koszinowski UH (2000). Viral mechanisms of immune evasion. Mol Med Today.

[17] Beachboard DC, Horner SM (2016). Innate immune evasion strategies of DNA and RNA viruses. Curr Opin Microbiol.

[18] Moreno-Altamirano MMB, Kolstoe SE, Sanchez-Garcia FJ (2019). Virus control of cell metabolism for replication and evasion of host immune responses. Front Cell Infect Microbiol.

[19] Mibayashi M, Martinez-Sobrido L, Loo YM, Cardenas WB, Gale M, Garcia-Sastre A (2007). Inhibition of retinoic acid-inducible gene I-mediated induction of beta interferon by the NS1 protein of influenza A virus. J Virol.

[20] Barral PM, Morrison JM, Drahos J, Gupta P, Sarkar D, Fisher PB, Racaniello VR (2007). MDA-5 is cleaved in poliovirus-infected cells. J Virol.

[21] Shi X, Wang L, Zhi Y, Xing G, Zhao D, Deng R, Zhang G (2010). Porcine reproductive and respiratory syndrome virus (PRRSV) could be sensed by professional beta interferon-producing system and had mechanisms to inhibit this action in MARC-145 cells. Virus Res.

[22] Wang D, Fang L, Li K, Zhong H, Fan J, Ouyang C, Zhang H, Duan E, Luo R, Zhang Z, Liu X, Chen H, Xiao S (2012). Foot-and-mouth disease virus 3C protease cleaves NEMO to impair innate immune signaling. J Virol.

[23] Barral PM, Sarkar D, Fisher PB, Racaniello VR (2009). RIG-I is cleaved during picornavirus infection. Virology.

